# Developing a Collaborative Agenda for Humanities and Social Scientific Research on Laboratory Animal Science and Welfare

**DOI:** 10.1371/journal.pone.0158791

**Published:** 2016-07-18

**Authors:** Gail F. Davies, Beth J Greenhough, Pru Hobson-West, Robert G. W. Kirk, Ken Applebee, Laura C. Bellingan, Manuel Berdoy, Henry Buller, Helen J. Cassaday, Keith Davies, Daniela Diefenbacher, Tone Druglitrø, Maria Paula Escobar, Carrie Friese, Kathrin Herrmann, Amy Hinterberger, Wendy J. Jarrett, Kimberley Jayne, Adam M. Johnson, Elizabeth R. Johnson, Timm Konold, Matthew C. Leach, Sabina Leonelli, David I. Lewis, Elliot J. Lilley, Emma R. Longridge, Carmen M. McLeod, Mara Miele, Nicole C. Nelson, Elisabeth H. Ormandy, Helen Pallett, Lonneke Poort, Pandora Pound, Edmund Ramsden, Emma Roe, Helen Scalway, Astrid Schrader, Chris J. Scotton, Cheryl L. Scudamore, Jane A. Smith, Lucy Whitfield, Sarah Wolfensohn

**Affiliations:** 1 Department of Geography, College of Life and Environmental Sciences, University of Exeter, Exeter, United Kingdom; 2 School of Geography and the Environment and Keble College, University of Oxford, Oxford, United Kingdom; 3 Centre for Applied Bioethics, School of Veterinary Medicine and Science, University of Nottingham, Leicestershire, United Kingdom; 4 Centre for the History of Science, Technology and Medicine (CHSTM), Faculty of Life Sciences, University of Manchester, Manchester, United Kingdom; 5 Biological Services, Health Schools, King's College London, London, United Kingdom; 6 Society of Biology, Charles Darwin House, London, United Kingdom; 7 Biomedical Services, University of Oxford, Oxford, United Kingdom; 8 School of Psychology, University of Nottingham, University Park, Nottingham, United Kingdom; 9 Joint Biological Services, College of Biomedical and Life Sciences, Cardiff University, Cardiff, United Kingdom; 10 Society of Biology, Charles Darwin House, London, United Kingdom; 11 TIK – Centre for Technology, Innovation and Culture, Faculty of Social Sciences, University of Oslo, Oslo, Norway; 12 Department of Geography, King's College London, London, United Kingdom; 13 Department of Sociology, London School of Economics, London, United Kingdom; 14 Institute of Pharmacology and Toxicology Department of Veterinary Medicine, Freie Universität Berlin, Berlin, Germany; 15 Department of Sociology, University of Warwick, Coventry, United Kingdom; 16 Understanding Animal Research, London, United Kingdom; 17 Centre for Research in Animal Behaviour, Psychology, University of Exeter, Exeter, United Kingdom; 18 Biological Services Facility (BSF), Faculty of Life Sciences, University of Manchester, Manchester, United Kingdom; 19 Department of Environmental Studies, Hobart and William Smith Colleges, Geneva, New York, United States of America; 20 Animal Sciences Unit, Animal and Plant Health Agency Weybridge, Addlestone, United Kingdom; 21 School of Agriculture, Food & Rural Development, Newcastle University, Newcastle upon Tyne, United Kingdom; 22 Exeter Centre for the Study of the Life Sciences (Egenis) & Department of Sociology, Philosophy and Anthropology, University of Exeter, Exeter, United Kingdom; 23 School of Biomedical Sciences, Faculty of Biological Sciences, University of Leeds, Leeds, United Kingdom; 24 Research Animals Department, Science Group, RSPCA, Wilberforce Way, Southwater, West Sussex, United Kingdom; 25 Biotechnology and Biological Sciences Research Council (BBSRC), Swindon, United Kingdom; 26 Faculty of Medicine & Health Sciences, University of Nottingham, Leicestershire, United Kingdom; 27 School of Planning and Geography, College of Art, Humanities and Social Sciences, Cardiff University, Cardiff, United Kingdom; 28 Department of the History of Science, University of Wisconsin—Madison, Madison, Wisconsin, United States of America; 29 UBC Animal Welfare Program, Vancouver, BC, Canada; 30 School of Environmental Sciences, University of East Anglia, Norwich, United Kingdom; 31 Faculteit of Law, VU University, Amsterdam, The Netherlands; 32 School for Social and Community Medicine, University of Bristol, Bristol, United Kingdom; 33 School of History, Queen Mary, University of London, London, United Kingdom; 34 Department of Geography and Environment, University of Southampton, Southampton, United Kingdom; 35 Honorary Research Associate, Geography Department, Royal Holloway, University of London, London, United Kingdom; 36 Department of Sociology, Philosophy and Anthropology, University of Exeter, Exeter, United Kingdom; 37 Institute of Biomedical and Clinical Sciences, University of Exeter Medical School, Exeter, United Kingdom; 38 Mary Lyon Centre, MRC Harwell, Harwell, United Kingdom; 39 Faculty of Science, The Open University, Milton Keynes, United Kingdom; 40 Named Veterinary Surgeons Group, Royal Veterinary College, London, United Kingdom; 41 School of Veterinary Medicine, University of Surrey, Guildford, Surrey, United Kingdom; Universidade do Porto Instituto de Biologia Molecular e Celular, PORTUGAL

## Abstract

Improving laboratory animal science and welfare requires both new scientific research and insights from research in the humanities and social sciences. Whilst scientific research provides evidence to replace, reduce and refine procedures involving laboratory animals (the ‘3Rs’), work in the humanities and social sciences can help understand the social, economic and cultural processes that enhance or impede humane ways of knowing and working with laboratory animals. However, communication across these disciplinary perspectives is currently limited, and they design research programmes, generate results, engage users, and seek to influence policy in different ways. To facilitate dialogue and future research at this interface, we convened an interdisciplinary group of 45 life scientists, social scientists, humanities scholars, non-governmental organisations and policy-makers to generate a collaborative research agenda. This drew on methods employed by other agenda-setting exercises in science policy, using a collaborative and deliberative approach for the identification of research priorities. Participants were recruited from across the community, invited to submit research questions and vote on their priorities. They then met at an interactive workshop in the UK, discussed all 136 questions submitted, and collectively defined the 30 most important issues for the group. The output is a collaborative future agenda for research in the humanities and social sciences on laboratory animal science and welfare. The questions indicate a demand for new research in the humanities and social sciences to inform emerging discussions and priorities on the governance and practice of laboratory animal research, including on issues around: international harmonisation, openness and public engagement, ‘cultures of care’, harm-benefit analysis and the future of the 3Rs. The process outlined below underlines the value of interdisciplinary exchange for improving communication across different research cultures and identifies ways of enhancing the effectiveness of future research at the interface between the humanities, social sciences, science and science policy.

## Introduction

A recent editorial in Nature makes the case that social, economic and cultural issues should be taken into account in the initial framing of research agendas as these factors are critical to the subsequent take-up of scientific developments [1]. The potential social, economic and cultural issues informing laboratory animal science and welfare are significant and complex. We review these below before outlining the methods and outcomes of a collaborative process for developing a future agenda for humanities and social scientific research on laboratory animal science and welfare. This process and resulting agenda aim to develop the capacity for future collaborative research involving the humanities and social sciences, to address these important issues and contribute to their inclusion in the framing of future research agendas in this field.

The use of animals in biomedical research continues to be an area of public and scientific debate. The broad social acceptability of laboratory animal research, as suggested in opinion polls in the UK [2], depends upon a tacit social contract between citizens, scientists and the state. Whilst individuals may oppose laboratory animal research, its continued social acceptability can be evidenced through these polls. Yet, they also indicate the conditionality of public support, showing how responses vary according to the extent to which there are no alternatives, minimisation of harms to animals, and benefits for human and/or animal health. This variability demonstrates the importance of assurances, assumed or demanded by different groups of the public, that the governance of research and practices of science can match these expectations. Relations between state, science and social trust are thus crucial to the social acceptability of laboratory animal research; yet, they are also contested and changeable [3–4]. Ideas about socially acceptable experimental practices involving laboratory animals have changed over time in response to changes within science and across society [5–8]. They also vary over space; evident in the recent European Citizens’ Initiative to ‘Stop Vivisection’ [9]. As the organisation of laboratory animal research becomes increasing transnational [10–11], with growing imperatives for translational benefits [12–14], and developing demands for transparency [15–18], the social relations underpinning support for laboratory animal research cannot simply be assumed. On the contrary, they should be taken into account in the framing of future research.

Social factors are also relevant to the policy interventions and internal practices of laboratory animal science and welfare. Social, economic and localized institutional factors influence the ability of those working within laboratory animal research and care to respond to new forms of regulation, ethical assessment, data practices and animal welfare science [19]. A growing number of policy processes are seeking to balance developments in biomedical research with considerations of animal welfare, for example through the international promotion of ethical review, harm-benefit analysis, application of the principles of the 3Rs (Replacement, Reduction and Refinement) [20] and the ARRIVE guidelines on reporting animal research [21–22]. Yet, these initiatives vary internationally and are often uneven or ambiguous in application [23–26], suggesting that culture has an important role to play. There are also efforts to extend care through international veterinary training [27], and harmonise regulations through policy review [28]; once again, these have to contend with and accommodate local differences in practice and social context. Furthermore, debates on reproducibility and bias, relevant to the benefits of laboratory animal research, indicate how individual, institutional and commercial pressures on scientists may influence the selection of data and presentation of results [29–33]. Given the importance of these and other social factors in shaping laboratory animal science and welfare, we propose a crucial role for humanities and social science research in developing evidence to understand the influence of social, economic, and cultural factors within the practices of laboratory animal science, as well as in the wider public.

This paper describes a collaborative process designed to create a shared research agenda for defining and prioritizing interdisciplinary questions around the social, economic and cultural dimensions to laboratory animal science and welfare. The process sought to define questions amenable to study by the concepts and methods of the humanities and social sciences and identify areas where scientists and other stakeholders agreed that innovative interdisciplinary approaches could be most productively applied. The process builds on recent experiments in the development of collaborative research agendas, which were pioneered in conservation biology and ecology [34–35], and have been extended to include research questions at the science-policy interface [36–37] and elsewhere [38]. Many of these have become both widely cited and generative of new research projects in their respective fields. As such, collaborative processes have been shown to contribute to capacity building for interdisciplinary enquiry by improving mutual understanding and trust between different research communities, especially at the interfaces of science and policy. This experiment in extending these processes to the development of a collaborative agenda for humanities and social scientific research on laboratory science and welfare confirms the value of framing research questions collaboratively through open dialogue and communication.

## Methods

The optimum process for structuring the production of a collaborative research agenda differs according to the aims of the study, the scope of the field and the scale of the enquiry [38]. The process used in this research had four main aims: to define a collaborative agenda for humanities and social scientific research on laboratory animal science and welfare, to enhance communication and understanding between disciplines, to develop relationships important for knowledge transfer and impact, and to increase research capacity within the social science and humanities. It followed prior methods in adopting a four-stage process consisting of the recruitment of participants, the generation of questions, the agreement of priorities (through discussion and voting), and the collective drafting of outcomes. At each stage, the process made explicit commitments to openness and inclusivity, in order to develop an honest and constructive dialogue between different perspectives in a field often characterised by polarized opinions. Previous initiatives on much broader topics have produced lists of up to 100 questions [34–38]. Our goal of producing 30 questions therefore reflects the more specific nature of the animal research topic, as well as our practical desire to maximise discussion within the time available. The methodology is outlined below; a more detailed explanation of every step used in this process is provided in S1 File.

The process was organised and facilitated by a small team of humanities and social science scholars. This group has experience of researching the social, historical and cultural dimensions to laboratory animal science and welfare [3, 17, 19, 39–45], and had previously collaborated in establishing the Laboratory Animals in the Social Sciences and Humanities (LASSH) network in 2014 [46]. These prior activities were an important precursor to building the relations, trust and networks for collaborative work. The organisers were also guided by past research on deliberative processes in controversial areas of science [47–48] and made explicit commitments to participants that the process would be inclusive, collaborative, deliberative and transparent. Inclusivity meant being aware of and open to the diversity of potentially relevant stakeholder perspectives, in recruitment and communication with participants. To facilitate a collaborative approach, the process sought to open-up established framings of the issues by a mix of methods: treating all submitted research questions anonymously, then allowing participants to refine questions through face-to-face deliberation and the exchange of reasons with others at the workshop. Transparency was maintained by informing participants of all stages of the process and in all iterations of the development and prioritization of research questions, via email and at the workshop.

The participants in this agenda-setting exercise were recruited through purposeful or theoretical sampling, rather than representative sampling. The aim is thus to maximise diversity in terms of the range of perspectives on laboratory animal science and welfare. The overall process involved 45 participants, with 35 attending the workshop, and incorporated a range of expertise from the humanities, social sciences, biological research, animal welfare science, science policy-makers, animal advocacy groups and other stakeholders (see author list). Around one third of those present were current personal licence holders, permitting them to carry out licensed procedures on animals under the UK’s Animals (Scientific Procedures) Act 1986, although a larger number had past experience of using animals in biomedical research. Each participant was encouraged to consult their colleagues and peers in generating the initial list of questions. Five participants reported running pre-workshops or discussion fora in their institutions. Around 100 individuals were involved in producing an initial list of questions, emailed to the organisers, indicating their proposed ideas for new interdisciplinary research on laboratory animal science and welfare.

The collated list of 136 questions was circulated to all participants, via email, for an initial round of voting on priorities. Participants then met at an interactive day workshop in London. This enabled participants to discuss and decide on the final agenda together, through a mix of small group discussions and plenary sessions. Small group discussions enabled the clarification of issues and the redefinition of questions, so they could be met by research in collaboration with the humanities and social sciences. The closing plenary involved discussion to prioritise these questions into a future agenda for new research on laboratory animal science and welfare. The final editing and grouping of questions took place over email. This resulted in a collaborative research agenda comprising 30 priority questions, grouped into four thematic categories to aid communication and application. No attempt was made to rank the final list of priority questions.

This exercise was considered and approved by the Geography Discipline Ethics panel for the grant holder, Gail Davies, at the University of Exeter. Other than protection of personal data, the research was not felt to raise significant ethical issues. All those participating in the submission and final definition of questions provided written consent to participate in the study. The workshop organisers, Davies, Greenhough, Hobson-West and Kirk, led on the production of the paper. All participants, by virtue of their contribution to generating, defining and prioritizing questions in the workshop, and via email, were invited to become authors of the paper.

## Results

The collaborative research agenda for humanities and social scientific research on laboratory animal science and welfare is presented below. The research questions produced reflect the considerable and collective efforts of all participants. Each question provides the starting point for developing future innovative research in the social sciences and humanities responsive to, and in dialogue with, the needs of the animal research and welfare community.

### Changing Contexts in Science and Policy

1 How are moves towards open science, data accessibility and greater transparency influencing research design and practices in laboratory animal research?
2 In what contexts do the practices and governance of animal research become responsive to change (e.g. in the context of new technologies and emerging risks), and how can these inform the development of better regulation?
3 What are the drivers for, and implications of, international circulations of expertise in relation to changing national practices and policies of laboratory animal science?
4 How does, and could, attending to animal welfare generate different forms of value (e.g. research innovations, economic opportunities, social acceptability) for different groups?
5 How is the credibility of animal models and non-animal alternatives constructed, decided upon and challenged in different contexts?
6 What factors (e.g. scientific, animal welfare, economic, political) influence the sourcing, breeding and transportation of animals in laboratory animal research and use?
7 In what ways have legislative categories that offer enhanced protection to some species over others, shaped and been shaped by attitudes to and uses of animals in research?
8 How do species categories and characteristics get used and amended as indicators of *sentience* within animal research and care practices?

### Cultures of Animal Care

9 How can a *culture of care* be defined, what does it look like in institutions where it is functioning well, and what factors enable or constrain its development?
10 How, and with what implications, does the practice and understanding of *a culture of care* differ according to personal, professional, institutional and other contexts?
11 How can animal care staff and other individuals be supported or empowered to improve good welfare practices and policy, and what are the institutional and other barriers to realising this?
12 What is the significance of *emotional labour*, and the potential for processes of de/sensitization, for developing a *culture of care* and sustaining animal care as a profession?
13 How can innovations in practices of care be fostered within and across local, national and international contexts?
14 How do recruitment strategies and motivations for entering the animal care profession impact upon a *culture of care*?
15 How do the emotional, embodied and affective relations between animals and people shape animal research and care practices?

### Public Attitudes and Engagement

16 Where are the opportunities for greater and meaningful public and stakeholder engagement in the policy and practices of animal research?
17 What, and in what contexts, do different publics want to know about animal research?
18 How do peoples’ life experiences and other factors (e.g. profession, religion, health, pet-keeping) influence attitudes and behaviours around animal research?
19 What factors influence the construction of trust around animal research in diverse publics?
20 What is the influence of primary, secondary and tertiary education on people's attitudes to the use of animals in education and research?
21 How do understandings of animal experience and personal motivation influence public attitudes towards the use of animals in research and how does this compare to other sectors (e.g. agriculture)?

### Ethical Review and Replacement, Reduction and Refinement (3Rs) in Animal Research

22 How do harm-benefit assessments of proposed animal research involve the contributions from different roles, knowledges and ethical positions, and how are these resolved in practice?
23 How is the promissory discourse around the *translation* of animal research to humans influencing practitioner, policy-maker and public understandings of harm-benefit analysis?
24 What are the consequences for laboratory animals, researchers and animal care staff of the new EU requirement to record the actual (as opposed to predicted) severity of procedures?
25 How do harm-benefits assessments vary according to the use of animals for different permissible purposes (e.g. basic research, treatment of disease, animal welfare, species preservation)?
26 What factors shape the format, content and communication of decision-making in the ethical review of animal research in different contexts?
27 In what ways have the 3Rs been taken up and interpreted in different national contexts?
28 What factors influence the way researchers in different types of organisations implement and use the 3Rs?
29 How do different stakeholders define, use, and prioritise the 3Rs, in both rhetoric and reality?
30 To what extent are the 3Rs still fit for purpose and in what ways might they need to be superseded or supplemented?

## Discussion

The final research agenda is a collective summation of current questions regarding the social, economic and cultural aspects of laboratory animal research and policy. We propose that this new agenda demonstrates the common ground on which future collaborative research can be developed. It can be used to ensure time and resources are directed to those issues commanding interest across the humanities and social sciences and where new research can make significant difference to laboratory policy and practice. We recognise there are barriers, especially in funding for interdisciplinary research in an increasingly competitive research environment. However, we suggest the collaborative derivation of this research agenda highlights the scientific, social and political value of this area of research, with topics closely aligned to funder priorities. For example, the UK’s BBSRC has recently established a collaborative network to foster the best in animal welfare research which involves social science and humanities scholars. Other examples of work which tie in to the agenda we describe here including work on data-driven biology and the 3Rs (BBSRC), the bioeconomy (Horizon 2010), big data and health innovation (ESRC). Together, these initiatives confirm the value of multi-disciplinary conversations which are increasingly central to research [49].

As we now discuss, the four themes listed above provide a broad framework for formulating research priorities and new programmes of research. First, there is an important set of questions which reflect the changing international landscapes of animal research. Research priorities here include understanding how international changes in biological research, open data and open access, legislation on the sourcing and use of animals, and understandings of sentience may alter the regulation and practice of animal research. Second, there are questions around the different aspects of a ‘culture of care’. The establishment and maintenance of a culture of care within institutions is now the explicit focus of regulation, training and compliance in the UK and EU. The research questions here suggest recognition of the growing importance of this concept, and reflect participant uncertainties around how it might be identified, understood and enacted across research and regulation. Thirdly, there is a recurrent interest in the ways different publics come to understand, trust and hold different attitudes towards animal research. These questions require consideration of changing cultural and social contexts, as well as the changing science and regulation of laboratory animal science and welfare. Finally, there is renewed attention and evaluation of the ethical framework underpinning animal research governance, including the principles of 3Rs (replacement, reduction and refinement) described by Russell and Burch’s [20]. Conceived in the 1950s, and coming to prominence from the 1990s, the 3Rs are now widely recognised as providing a framework for minimizing suffering within laboratory animal practice. Yet, there are challenges in their implementation, and questions about their continued applicability. There is also recognition that there are aspects of ethical review that exceed the 3Rs, such as good reporting, reproducibility and robust experimental design [50], and also questions about the assumptions involved in harm-benefit assessment, which are all open to further interdisciplinary enquiry.

The derivation of this research agenda through communication across the humanities, social and laboratory animal sciences demonstrates the potential for developing collaborative responses to these questions. It also acts as further validation of this collaborative method was has previously been used in other fields [34–38]. Crucially in our case, there was a clear commitment from the spectrum of participants to ways of working which were open-minded, transparent and accountable. Meeting face-to-face, and over time, helps build communities of trust across different disciplines and perspectives. This is crucially important given animal research often involves entrenched positions. It also helped create a safe space where, for example, junior technicians spoke openly in the presence of management and policy makers. The combination of individuals and interests in this exercise allowed questions to emerge in novel ways, supported by evidence from practitioners and enriched by interdisciplinary exchange. This ensured no one discipline dominated the final framing of questions, and that questions have both relevance for the scientific community and significance for researchers within the humanities and social sciences.

Yet, the disciplines involved in this process do have specialised languages reflecting the concepts and practices important to them [51–52]. There are differences across and within the sciences, social sciences and humanities. The involvement of laboratory animal scientists and other practitioners was essential for framing questions with the potential to gain traction with stakeholders. The involvement of these participants meant others could clarify their understandings of key terms, roles and concepts in laboratory animal science at an early stage. Yet, some ambiguities could not be removed from the final questions. For example, a good ‘culture of care’ is now a key objective in the regulation of laboratory animal research in and beyond the UK [53]. Yet, the term has wider meanings in clinical contexts [54], in relation to care ethics [55], or in relation to other concepts such as emotional labour [56]. We have left certain terms in italics to indicate their potential variability. However, we have not sought to remove these ambiguities as they could be productive—in signalling adaptability and opening up useful conversations—or a challenge—in indicating an inconsistency which is an obstacle to communication. Both are significant points for further research. In addition, and across all questions, technical discussion explored whether questions were addressed to research on whole organisms, or research using animal tissues. We would encourage future users of this agenda to identify and draw out these differences when relevant.

The involvement of representatives from anthropology, geography, history and sociology foregrounds an interest in social and spatial variations in laboratory animal practices. This was also evident in practitioner enquiries into international and other differences, their causes and implications for laboratory animal science and welfare. Some geographical issues are explicit in the final set of research priorities, but going forward we would emphasize the need for empirical studies across laboratories and across countries to fully understand the increasingly globalized contexts of many of the questions. Contribution from historians and humanities scholars also highlighted how relations between laboratory animal science, animal welfare and the governance of research have changed over time. These conversations were similarly enriched by personal accounts from those with long careers in animal research and welfare. Current research policies and practices have histories that are important for understanding the circumstances in which they emerged, their present operation and future development. Some research questions inquire into particular aspects of history, but again there is an opportunity to add a temporal dimension to other aspects of this agenda. Throughout, this attention to comparison foregrounds the interactions between regulatory frameworks, policy processes and the implementation of practice, which are often absent from individual ethnographic accounts of animal care.

The emergence of new research ideas through this process strengthens studies suggesting humanities and social science scholars can make important contributions by facilitating reflection on scientific practices within, as well as outside of, the scientific community [57–59]. This approach to science does not seek to undermine the value of scientific knowledge, but to recognise its plurality in practice and identify the contextual factors which influence how different ways of knowing and working with animals emerge as dominant in different times and places [60]. It also emphasizes the need to foster dialogue about the diversity of practices across sites, to help identify and share best practice, and to understand what enables or constrains multi-disciplinary communication and collaboration, without collapsing one discipline into another.

The ongoing nature of social, economic and cultural change means it is unlikely there will be a simple or final answer to the research questions generated in this collaborative agenda-setting process. For experimental scientists, working to generate data and reduce uncertainty, the open and reflexive nature of questioning and explanation in the humanities and social sciences can be challenging. Nevertheless, this was not the dominant experience in this exercise. The collaborative process and publication demonstrates the shared commitment to communication and research across disciplinary divides. By staging a structured conversation to generate research questions together, this process has deepened interdisciplinary understandings and demonstrated future capacity for careful collaborative enquiry.

## Conclusions

To recap the *Nature* editorial with which we opened, we would agree we ‘need to support a capacity to understand society that is as deep as […] our capacity to understand the science’ in the area of laboratory animal science and welfare [1]. To achieve this, we need to generate and prioritise research questions that effectively get to the heart of the social and ethical issues, and adequately address the dilemmas and challenges faced by laboratory animal stakeholders. The authors consider that the questions resulting from this interdisciplinary process do have significant merit in functioning as a credible research and funding agenda going forward. This agenda should therefore encourage future empirical research projects which demonstrate the social, economic and cultural interactions that influence responses to new scientific research and regulation, within and outside of the scientific community. Indeed, the questions identified in this collaboration are already being used by some of the authors to develop novel research proposals and deepen relationships for shared enquiry. We therefore predict that future social science research will be able to provide greater understanding of how biomedical research, using animals, succeeds or fails to become credible with the public. Policy relevant work could complement welfare science agendas focusing on the experience of the animal by identifying the international and local infrastructures that influence the adoption of particular practices. Humanities research can contribute to recognising the communicative, embodied and empathetic practices that underpin a ‘culture of care’ and connect the day-to-day work of laboratory animal research and welfare with the welfare of staff and researchers. More broadly, interdisciplinary agenda-setting processes of the kind described in the present paper can help secure advances in our understanding of contested areas of scientific and technological practice.

## Supporting Information

S1 File Methodological Details. (DOCX)
