# Supplementary material for: Developing a Collaborative Agenda for Humanities and Social Scientific Research on Laboratory Animal Science and Welfare
Source: PLoS One. 2016 Jul 18;11(7):e0158791. doi: 10.1371/journal.pone.0158791 (PMC4948886; doi:10.1371/journal.pone.0158791)
Supplement: S1 File — (DOCX) [file pone.0158791.s001.docx]

# **S1 File: Methodological Details**

The aim of generating priority research questions for the social and historical sciences of value to furthering laboratory animal research and welfare led to the decision to use purposive sampling for recruitment, and influenced the methods used to generate and refine questions. Purposive sampling involves deliberately inviting participation from those known to have a stake or interest in the issues. Participant recruitment took place through the dissemination of personal invitations, via research networks and contacts, and the distribution of information, via relevant research and practitioner mailing lists (e.g. the Laboratory Animals Science Association (LASA)). The details of the process were further communicated through the LASSH network website (http://labanimalstudies.net/). All who enquired were fully informed of the aims of the exercise, and, if they were interested, invited to contribute to the process.

The final 45 participants had different interests in laboratory animal welfare. These both reflect the relevant stakeholders in this area (researchers, animal technologists, Named Veterinary Surgeons, science promoters and animal welfare advocates) and represent the potential audiences for the agenda-setting exercise (policy-makers, funders, natural and social researchers). Around half the participants were researchers working in the social sciences and humanities on issues around animals or science policy, and half were working within the communities of policy and practice in the laboratory animal sciences, including scientific researchers, animal technicians, named veterinary surgeons, animal welfare groups, and learned societies. The meeting attracted international participation, with contributors from Canada, the USA, the Netherlands, Norway and Germany, as well as across the UK, demonstrating the growing interest and scope for transnational research in this area. Participant details were circulated at the workshop, and, for those who consented, posted online and as well as included in the author list for this publication.

Participant recruitment strategies specifically included members of private industry, through personal contacts and gatekeepers. However, no one currently active in this community took part, although some participants had previous experience in the sector. This is a gap, which is likely to result in absences in the issues included in this research agenda and could be addressed in future work. However, it does not undermine the value and importance of the collaborative research priorities here. Indeed, one value of this proposed programme of research is that it is able to act as a starting point for future conversations with further stakeholders, for example, industry experts or those based outside the EU.

Each participant was encouraged to consult their colleagues and peers in generating the initial list of questions. Five participants reported running pre-workshops or discussion fora in their institutions. Not all reported exactly how many they engaged, but it is estimated that around 100 individuals were involved in producing the initial list of questions. Each participant was invited to submit 3-6 initial questions, but no absolute limit was imposed. In total, 136 questions or issues were suggested at this stage. These were collated into 6 themes with approximately equal numbers of questions by the organisers. Similar questions were grouped to aid the identification of overlaps and the organisation of discussions. The collated list was circulated to all participants, via email, for an initial round of voting on priorities. Each participant was given 10 votes to allocate to the issues they considered the most important. Contributors were also invited to suggest alternative wordings, favoured wordings from similar questions, alternative groupings for questions, and questions which could be merged. Nineteen questions, often duplicates or questions of factual information, received no votes at this stage. The results of the first round of voting were circulated to all participants, with the qualification that these were indicative only, aiming to help participants gauge and engage the spread of opinion on different issues at the workshop.

Discussion, selection and refinement of the research questions took place at a one-day workshop in London in October 2014. Not all who participated in the first stage were able to join the final group of 35 at the workshop, though two individuals did participate by video conferencing. The workshop opened with an introduction to the aims and process, explaining the focus and format of research questions in the humanities and social sciences, and included the opportunity for comments and clarifications on the process. Participants signed up for one of six parallel discussion groups organised around: Research Infrastructures, Incentives and Innovations; Public Engagement, Education and Participation; Reporting and Evaluating Harms and Benefits; The Application and Development of the 3Rs; Ways of Knowing Laboratory Animals; Animal Care Taking and Cultures of Care.

Each small group was composed of a mixed disciplinary membership and was chaired by a humanities or social science scholar to help frame questions in a format relevant for this exercise. Members of each group received paper copies of the submitted questions, voting patterns and the detailed submitted comments for their theme. Over a two-hour period they were asked to discuss the full set of issues, then propose their four key questions and two reserve questions for consideration in the final plenary. A rapporteur kept a record of the discussions and a list of the refined questions. The small groups approached the task of focusing their questions in slightly different ways. Some worked on the most popular questions first. Some started by eliminating the questions with no votes. Others sought to reframe the full range of issues into six inclusive questions. In each group, either the rapporteur or chair had prior experience of deliberative processes, so as to support the consensus-based discussion of the final questions. In addition, either the rapporteur or chair was external to the main organising team, so as to strengthen diversity and openness. The rapporteurs electronically transcribed the questions in the lunch break for the afternoon session.

In the final plenary session, the lead author led a collective discussion of the results, with each small group invited to talk through their experience and present their priority and reserve questions to the workshop. Collective revision of the developing research agenda was made possible by projecting text on a screen, allowing questions and comments from all participants to be incorporated and agreed in real time. This enabled the merging of some questions, as well as the refinement, clarification and further justification of others. The workshop raised important issues around vocabulary, the meanings implied by the languages used by different disciplines, and their implications for engaging different research communities. The final editing of questions took place over email due to time constraints on the day. This also sought to be consensus-based, with a full written record of the revisions and rationale for changes circulated to all participants at each iteration. The final editing put questions into a consistent format, whilst retaining the ‘spirit’ of the question, and clarified grammar and language, whilst recognising there were differences between disciplines and individuals. Whilst there are still some overlaps across the topics, the specific manner in which each question is asked is distinctive and represents an important issue for participants.
